# Long non-coding RNA LINC01314 represses cell migration, invasion, and angiogenesis in gastric cancer via the Wnt/β-catenin signaling pathway by down-regulating KLK4

**DOI:** 10.1186/s12935-019-0799-9

**Published:** 2019-04-11

**Authors:** Lin Tang, Jian-Bo Wen, Ping Wen, Xing Li, Min Gong, Qiang Li

**Affiliations:** Department of Gastroenterology, Jiangxi Pingxiang People’s Hospital, No. 8, Wugongshan Middle Road, Pingxiang, 337000 Jiangxi People’s Republic of China

**Keywords:** Long non-coding RNA LINC01314, Kallikrein 4, Wnt/β-catenin signaling pathway, Angiogenesis, Gastric cancer

## Abstract

**Background:**

In recent years, gastric cancer (GC) has become a major cause of mortality among various malignancies worldwide with high incidence rates. Long non-coding RNA (lncRNAs) may serve as oncogenes and tumor suppressors in cancers. Therefore, we investigated the effect of LINC01314 on the development of GC cells in relation to the Wnt/β-catenin signaling pathway.

**Methods:**

Microarray data analysis was conducted to screen GC-related differentially expressed lncRNAs, followed by determination of the binding interaction between LINC01314 and kallikrein 4 (KLK4). Human GC cell line SGC-7901 was treated with over-expressed or silenced LINC01314 or KLK4 to investigate the mechanism LINC01314 affecting GC cellular activities. The levels of KLK4, Wnt-1, β-catenin, cyclin D1, N-cadherin and E-cadherin were measured, and cell invasion and migration were evaluated. Next, the tumor weight, micro-vessel density (MVD) and the expression of VEGF-C and VEGFR-3 in transplanted tumors were measured.

**Results:**

LINC01314 was poorly expressed in GC cells and KLK4 was revealed to be a direct target gene of LINC01314. Overexpressed LINC01314 or silencing of KLK4 led to inhibited GC cell migration and invasion, corresponding to decreased Wnt-1, β-catenin, cyclin D1 and N-cadherin while increased E-cadherin. Also, in response to over-expression of LINC01314 or silencing of KLK4, tumor weight and the MVD of transplanted tumors were reduced and angiogenesis was suppressed, which was indicated by down-regulated positive expression of VEGF-C and VEGFR-3.

**Conclusion:**

The findings indicated that over-expression of LINC01314 down-regulated KLK4 to inhibit the activation of the Wnt/β-catenin signaling pathway, thus suppressing migration, invasion, and angiogenesis in GC cells, which provides new insight for the treatment of GC.

## Background

Gastric cancer (GC) is one deadliest cancer all around the world, constituting for a quarter of all carcinoma occurrence rates across China [1]. It is also the chief cause of cancer-related deaths, and estimations suggest that 400,000 new diagnosis and 300,000 deaths occur every year, in China [2]. According to an epidemiology study on GC, lifestyle and environmental factors are highly associated to the etiology of the disease [3]. While the worldwide occurrence of GC has significantly reduced and the long-term survival rate has improved dramatically in the past decades, it remains to be a crucial contributor to the global cancer burden [4]. GC also has high reoccurrence rate, and 25% post-operative patients may develop lymph node metastasis (LNM) [5]. Moreover, angiogenesis is essential for tumor survival and growth, as establishment of new micro-vessels provides nutrition for tumor cell proliferation [6]. Therefore, the search for novel therapeutic modalities that targets GC has become a pressing concern in order to raise the quality of life of patients plagued by GC [7].

Long non-coding RNAs (lncRNAs) are part of a diverse class of newly characterized lncRNAs that play imperative regulatory functions in genes expressed during the development and differentiation of multicellular organisms [8]. These lncRNAs have been emphasized as new players in tumor development either as oncogenes or suppressors of tumorigenesis, or both in certain conditions [9]. For instance, new lncRNAs (i.e. H19, MEG3, PVT1, BANCR and FER1L4) have been verified as oncogenes or anti-oncogenes of GC [10]. Similarly, LINC01314 suppresses the progression of hepatoblastoma via regulation of the Hippo-YAP signaling pathway [11]. Based on the online prediction website, kallikrein 4 (KLK4) was predicted to be a target gene of LINC01314. KLK4 is a member of the family of 15 human kallikrein-related peptidase of trypsin-like serine proteases [12, 13]. KLK4 expression has been revealed to be correlated to cellular progression of prostate cancer, and its elevation proposes KLK4 as a promising biomarker for the immune intervention [14]. Cui et al. recently demonstrated that KLK4 could regulate the Wnt/β-catenin signaling pathway to influence the development of oral squamous cell carcinoma (OSCC) [15]. Recently, the Wnt/β-catenin pathway was reported to be essential for the maintenance of GC stem cell population [16], and a study showed its crucial role in the self-renewal of cancer stem-like cells in human GC [17]. Aberrant Wnt/β-catenin signaling activation and lncRNAs expression are then greatly linked to tumor progression, with the former abnormally triggered by the defective activity of the latter [18, 19]. Lastly, recent work has shown that a candidate lncRNA, CASC2, negatively regulates the Wnt/β-catenin signal cascade by reducing the β-catenin expression and overriding downstream processes targeted by the Wnt/β-catenin signaling pathway during bladder cancer tumorigenesis and progression [20]. These findings suggested a hypothesis that LINC01314 might act as a potential tumor suppressor in GC via the Wnt/β-catenin signaling pathway. Thus, the current study aims to explore the role of LINC01314 in its potential effects on migration, invasion and angiogenesis of GC cells with the involvement of Wnt/β-catenin signaling events.

## Materials and methods

### Microarray data analysis

GC chip data (GSE19826) and annotation probe file were obtained from the Gene Expression Omnibus (GEO) database (http://www.ncbi.nlm.nih.gov/geo), with the chips detected using the Affymetrix Human Genome U133 Plus 2.0 Array. Background correction and normalization for chip data were performed using the Affy installation package of R software [21]. Next, the Linear models and Empirical Bayes Methods of the Limma installation package as well as traditional *t*-test forms were applied to perform a nonspecific filtration process for gene expression data to screen differentially expressed lncRNAs [22]. The differentially expressed lncRNA was predicted using the ment Matrix (MEM, http://biit.cs.ut.ee/mem/) website. MEM is a web-based tool used to perform co-expression queries on large collections of gene expression experiments, which offers opportunities to several hundreds of publicly available gene expression datasets of different tissues, diseases and conditions, arranged by the species and microarray platform types [23]. Kyoto Encyclopedia of Genes and Genomes (KEGG) enrichment analyses of target genes were performed by WebGestalt database (http://www.webgestalt.org) to identify the major biochemical metabolic pathways and signaling pathways involved in the target genes [24].

### Cell culture

Human GC cell line SGC-7901 obtained from the cell bank of the Institute of Biochemistry and Cell Biology of Shanghai Institute of Life Sciences of Chinese Academy of Sciences (Shanghai, China) was thawed from the cryostat, immediately placed in water bath at 37 °C with the cryovial. The cryovial was shaken to revive the cells which were then pipetted into new centrifuge tubes. Next, the cells were resuspended in bovine serum-free medium RPMI1640 (PM150110, Procell Life Science and Technology Co., Ltd., Wuhan, China), and centrifuged at 403×*g* for 5 min with supernatant removed. Following this, the collected pellets of cells were washed once, and then resuspended with the culture medium containing 15% fetal bovine serum (FBS) and seeded in culture flasks. The setup was incubated at 37 °C in 5% CO_2_ and then sub-cultured as necessary. Cells at the logarithmic phase of growth were obtained for further experimentation.

### RNAi expression vector construction

The siRNA sequences of LINC01314 and KLK4 were designed using the siRNA design software of Ambion (Austin, Texas, USA). The base sequences of both sides of the Loop (TTCAAGAGA) site were complemented, and the LINC01314 and KLK4 shDNA templates were prepared. Next, HindIII and BamHI sticky restriction sites were added to each end of the shDNA template and were corresponded to restriction sites on both sides of pSilencer 4.1-CMV neo (AM5779, Ambion, Texas, USA). The target genes and the vector were connected by Ligase 4 and then transformed to DH5α competent cell. The genes were marked and positive clones were screened.

### Bioinformatic prediction and dual-luciferase reporter gene assay

The biological prediction website RNA22 (https://cm.jefferson.edu/rna22/) was employed in order to analyze the target gene of LINC01314 and verify whether KLK4 was a direct target gene of LINC01314. Subsequently, the target sequence and mutant (Mut) sequence were designed based on the binding site between KLK4-mRNA-3′-untranslated region (UTR) and LINC01314. Next, the target sequence was chemically synthesized, during which restriction sites *Xho*I and *Not*I were inserted into the ends of the sequence respectively. The synthesized target fragment was then cloned into the PUC57 vector. After identification of positive cloning, recombinant plasmids were identified using DNA sequencing, sub-cloned into the vector, and transformed into escherichia coli DH5α cells to amplify the plasmids. Following that, the plasmids were extracted according to the instructions of the Omega small-quantity extraction kits. Cells were then inoculated into a 6-well plate at the density of 2 × 10^5^ cells/well. Upon adhering to the wall, the cells were treated following the aforementioned methods, after which the cells were cultured for 48 h and collected. In strict accordance with the protocols of dual luciferase detection kits (Genecopoeia, Rockville, MD, USA), the effects of LINC01314 on the luciferase activity of KLK4-3′-UTR were detected, and the fluorescence intensity was measured using the Glomax20/20 luminometer (Promega, Madison, WI, USA).

### Cell grouping

Human GC cell line SGC-7901 at the logarithmic phase of growth was seeded in a 6-well cell culture plate (2 × 10^5^ cells/well), and treated when the cells adhered to the wall and reached 60%–80% confluence. The experiment was performed in line with instructions of lipofectamine 2000 kit (11668-027, Invitrogen, Calsbad, CA, USA). A total of 250 μL serum-free medium RPMI1640 was used to dilute 100 nM DNA sequences (final concentration was 50 nM), followed by mixing and incubation for 5 min. Another 250 µL serum-free medium was used to dilute 5 µL lipofectamine 2000, followed by mixing and incubation for 5 min. Next, the above two mixtures were incubated for 20 min, added to the wells, and cultured at 37 °C in 5% CO_2_ and saturated humidity. After 4–6 h, the medium containing transfection solution was removed, and then the cells were re-cultured using fresh RPMI1640 containing 10% FBS for 24–48 h for further experimentation. The cell lines were then classified in the following groups: the blank group (without any treatment), the negative control (NC) group (treated with universal scramble siRNA), the si-LINC01314 group (treated with LINC01314 siRNA plasmids), the OE-LINC01314 group (treated with LINC01314 plasmid), the si-KLK4 group (treated with KLK-4-siRNA plasmid), the OE-LINC01314 + si-KLK4 group (treated with LINC01314 plasmid + KLK4 siRNA plasmid), and the OE-LINC01314 + LiCl group (treated with LINC01314 plasmid + specific activator of Wnt/β-catenin signaling pathway). All aforementioned sequences were purchased from Shanghai GenePharma Co., Ltd. (Shanghai, China).

### RNA isolation and quantitation

Cells from each group were collected, and total RNA was extracted using Trizol one-step method following manufacturer’s guidelines (15596-018, Invitrogen, Carlsbad, California, USA). The collected RNA was resuspended in diethyl pyrocarbonate (DEPC)-treated ultrapure water (A100174-0005, purchased from Sangon Biotech Co., Ltd., Shanghai, China). Next, the ND-1000 UV/visible spectrophotometer (Thermo Fisher Scientific, Massachusetts, USA) was used to check total RNA quality as well as to quantify and standardize RNA concentration between the groups. The reverse transcription of the extracted RNA was performed by the two-step method following the instructions of the kit (Thermo Fisher Scientific, Massachusetts, USA). The complementary DNA (cDNA) obtained by reverse transcription was temporarily stored at − 80 °C. Subsequently, reverse transcription quantitative polymerase chain reaction (RT-qPCR) was performed using the TaqMan probe method, and the reaction system was conducted as per kit’s protocol (KR011A1, Beijing Puyihua Technology Co., Ltd, Beijing, China). The sequences of the primers used are shown in Table 1. RT-qPCR instrument (Bio-Rad iQ5, Bio-Rad Laboratories, San Francisco, USA) was used for the detection of amplification. Glyceraldehyde-3-phosphate dehydrogenase (GAPDH) served as the internal reference. The 2^−∆∆Ct^ method was employed to determine the levels of gene expression. 2^−∆∆Ct^ represented the ratio of target gene expression between the experiment group and blank group using the following formula: ΔΔCT = ΔCt _experiment group _− ΔCt _blank group_, where ΔCt = Ct _target gene_ − Ct _GAPDH_ [25].

**Table 1:** Primer sequence for RT-qPCR

Gene	Primer sequence (5′–3′)
LINC01314	F: TGGGGCTGCCCTGGCCCGAGGGAC
	R: CCAGTGCAGGGTCCGAGGT
KLK4	F: ATCTGCCTACCTCCGGAGTC
	R: TTAACTGTCCTGGATGGTTTT
Wnt-1	F: GCTAGCGAAAGTCATTGGC
	R: CATTGCATCGAAGTCAGTG
β-catenin	F: GCTAGCTAAGTCAGTCGGG
	R: CGTATATAGCTAGCTGGCTA
cyclin D1	F: AAGCCTCAGCCCTCCCCAGCTGCCAG
	R: AACCAACAACAAGGAGGATG
N-cadherin	F: TTTGATGGAGGTCTCCTAACACC R: ACGTTTAACACGTTGGAAATGTG
E-cadherin	F: CGAGAGCTACACGTTCACGG
	R: GGGTGTCGAGGGAAAAATAGG
GAPDH	F: CGGAGTCAACGGATTTGGTAT
	R: AGCCTTCTCCATGGTGGTGAAGAC

*RT-qPCR* reverse transcription quantitative polymerase chain reaction, *F* forward, *R* reverse, *N-cadherin* neural cadherin, *E-cadherin* epithelial cadherin, *GAPDH* glyceraldehyde-3-phosphate dehydrogenase, *KLK4* kallikrein 4

### Western blot analysis

Cells (2 × 10^5^ cells/well) from each group were collected in centrifuge tubes, and then added with 100 μL Radio Immunoprecipitation Assay (RIPA) lysis buffer (R0020, Beijing Solarbio Science and Technology, Co., Ltd, Beijing, China) containing 1 mmol/L phenylmethanesulfonyl fluoride. Next, the cells were centrifuged at 1610×*g* until homogenously lysed, allowed to settle in an ice bath at 4 °C for 30 min and then centrifuged at 25,764×*g* for 4 min. The supernatant was collected and preserved at − 80 °C until further processing. Total protein content from the supernatant was extracted and protein concentration was tested using a bicinchoninic acid (BCA) kit (AR0146, Wuhan Boster Biological Technology, Ltd, Wuhan, China). The concentration of each sample was adjusted to 3 μg/μL. After being added with the loading buffer, extracted protein was boiled at 95 °C for 10 min with 30 μg proteins added to each well, and then separated with 10% sodium dodecyl sulfate polyacrylamide gel electrophoresis (SDA-PAGE) and then blotted to polyvinylidene fluoride (PVDF) membrane (P2438, Sigma company, USA) using the semi-dry electric transfer membrane method. Next, the membrane was sealed for 1 h with 5% bovine serum albumin (BSA), added with primary antibody mouse antibodies KLK4 (ab71234, dilution ratio of 1:500), Wnt-1 (ab105740, dilution ratio of 1:250), β-catenin (ab22656, 2 µg/mL), cyclin D1 (ab139260, dilution ratio of 1:1000), N-cadherin (ab98952, dilution ratio of 1:1000), E-cadherin (ab1416, dilution ratio of 1:50) and GAPDH (ab181602, dilution ratio of 1:10,000) at 4 °C overnight. Following this, the membrane was washed with Tris-buffered saline with Tween 20 (TBST), and added with the corresponding goat anti-mouse secondary antibody (ab6789, dilution ratio of 1:2000), followed by incubation for 1 h. All aforementioned antibodies were from Abcam Inc. (Cambridge, MA, USA). Lastly, the membrane was washed with a chemiluminescent reagent, and then viewed under a Bio-rad Gel Dol EZ imager (GEL DOC EZ IMAGER, Bio-rad, California, USA). The intensity of the grey target band was analyzed using the ImageJ software.

### Transwell assay

Matrigel (40111ES08, Shanghai Yisheng Biotech Co., Ltd., Shanghai, China) was dissolved overnight at 4 °C and diluted with serum-free Dulbecco’s modified eagle’s medium (DMEM) culture medium at the ratio of 1:3. Next, 30 μL diluted Matrigel was coated in the apical chamber of the transwell chambers three times, every 10 min (15 μL, 7.5 μL, and 7.5 μL, respectively), so that Matrigel would be evenly applied to all wells of the apical chamber. At 48 h after treatment, the SGC-7901 cells were collected to prepare cell suspension. Then, the cells were seeded in the apical chamber, and 0.5 mL DMEM culture medium containing 10% FBS was added to the 24-well basolateral chamber followed by incubation for 48 h at 37 °C with 5% CO_2_. Following this, the non-penetrating cells in the apical chamber were gently wiped off using cotton swabs. Next, the membrane was fixed with 95% ethanol for 15–20 min. Afterwards, the membrane was stained with methylrosanilinium chloride for 10 min and subsequently rinsed with clean water. A high-magnification inverted microscope was used for observation. A total of 5 high power fields were randomly selected from each sample to calculate the mean value of cell number. The number of cells in each group that penetrated the Matrigel was used as an index to evaluate cell invasion. The experiment was repeated 3 times to obtain the mean value.

### Scratch test

SGC-7901 cells were collected and then inoculated into a 6-well plate with cell density of 1 × 10^5^ cells per well. When cell confluence reached 90%, a 200 μL pipette tip was used to scratch the well plate four times horizontally and vertically. The width of the scratches was measured to calculate the healing rate of each group [the scratch width at 0 h − the scratch width at 24 h)/the scratch width at 0 h × 100%]. The experiment was repeated 3 times to obtain the mean value, and compare the cell migration in each group.

### Immunofluorescence

SGC-7901 cells at the logarithmic phase of growth were selected and grew on a processed coverslip. After undergoing aforementioned experimental treatments, the cells were fixed using 4% paraformaldehyde and ruptured with 0.2% Triton X-100. Next, the cells were added with 5% BSA blocking solution at 37 °C for 30 min. Following this, the blocking solution was removed, and the cells were added with the corresponding rabbit antibodies, N-cadherin (ab18203, 5 µg/mL, Abcam Inc., MA, USA) and E-cadherin (ab1416, 5 µg/mL, Abcam Inc., MA, USA). The cells were then incubated overnight, added with Texas red-labeled secondary antibody at 37 °C for 30 min avoiding exposure to light. Lastly, the cells were stained with 4′,6-diamidino-2-phenylindole (DAPI), sealed with glycerin and observed and photographed under an inverted laser confocal microscope.

### Xenograft tumor in nude mice

The subcutaneous xenograft tumor experiment was conducted as follows: 50 BALB/c nude mice (aged 5 weeks with weight of 18–22 g) (J004, Nanjing Better Biotechnology Co., Ltd., Nanjing, Jiangsu, China) were raised under specific-pathogen-free (SPF) grade conditions. The mice were fed sterilized food at 18–22 °C with 50–60% humidity, less than 20 ppm of ammonia concentration and frequency of ventilation of 10–20 times/h. The nude mice were randomly grouped into the blank group (nude mice injected with SGC-7901 cells without any treatment), the NC group (nude mice injected with SGC-7901 cells treated with scramble siRNA), the si-LINC01314 group (nude mice injected with SGC-7901 cells treated with LINC01314 siRNA plasmid), the OE-LINC01314 group (nude mice injected with SGC-7901 cells treated with LINC01314 plasmid), the si-KLK4 group (nude mice injected with SGC-7901 cells treated with KLK4 siRNA plasmid), the OE-LINC01314 + si-KLK4 group (nude mice injected with SGC-7901 cells treated with LINC01314 plasmid + KLK4 siRNA plasmid), and the OE-LINC01314 + LiCl group (nude mice injected with SGC-7901 cells treated with LINC01314 plasmid + Wnt/β-catenin signaling pathway specific activator). Each group comprised of 10 nude mice, with 5 males and 5 females. The cells treated for 24 h were detached with trypsin in order to prepare a cell suspension with cell density to 1 × 10^5^ cells/mL. Following this, the local skin of nude mice was disinfected, and 0.5 mL cell suspension was injected into the subcutaneous area of right forelimb scapular of each mouse. When the cell suspension was injected, a syringe needle was lightly tapped further into the skin and then gently lifted. After the needle was pulled out, the local skin was held down for 30 s to prevent leakage of cell suspension. The nude mice were raised to observe its overall condition as well as the condition of the inoculation site. All nude mice were sacrificed after 5 weeks post inoculation, and the gross tumor specimens and the tumor weight were observed and measured respectively.

### Immunohistochemistry

Tumor specimens were selected, fixed in neutral formalin, dehydrated and then embedded in paraffin. Then the specimens were sliced into 5 μm serial sections, and subjected to conventional dehydration. Next, the sections were treated with 3% hydrogen peroxide for 10 min, thermally repaired with antigen, and then blocked with normal non-immune animal serum for 20 min. Afterwards, the sections were added with the primary antibody cluster of differentiation 34 (CD34) rabbit polyclonal antibody (dilution ratio of 1:200), vascular endothelial growth factor (VEGF)-C rabbit polyclonal antibody (dilution ratio of 1: 50) and VEGF receptor (VEGFR)-3 rabbit polyclonal antibody (dilution ratio of 1:100) and then incubated at 4 °C overnight with phosphate buffered saline (PBS) serving as the negative control. With the addition of the biotin-labeled secondary antibody (goat anti-rabbit IgG), the sections were then incubated at 37 °C for 20 min. After that, the sections were stained with diaminobenzidine (DAB), counterstained with hematoxylin, and then dehydrated, cleared, mounted and observed under a microscope. The number of micro-vessels in 10 fields was counted, and the mean value represented the average micro-vessel density (MVD) according to the expression of CD34.

### Statistical analysis

Statistical analyses were performed using the SPSS 21.0 software (IBM Corp. Armonk, NY, USA). Measurement data were expressed as mean ± standard deviation. Comparisons between two groups were analyzed using the *t*-test, while multiple groups were compared by one-way analysis of variance (ANOVA). Non-parametric data were expressed either as percentage or rate and compared with the Chi square test. Data for multiple groups were compared using ANOVA tested for homogeneity of variance. If there was no significant difference in variance analysis, the q-test was further employed to make a pairwise comparison. Meanwhile, when there was heterogeneity of variance, non-parametric rank sum tests were used instead. Test level was set at α = 0.05, and *p *< 0.05 was considered to be statistically significant.

## Results

### Verification of KLK4 as a direct target gene of LINC01314

**Fig. 1:**
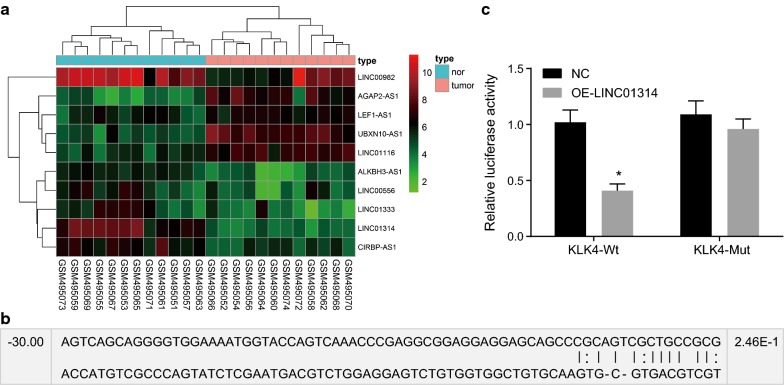
LINC01314 was identified to target the KLK4 gene. **a** Heat map of differentially expressed lncRNAs associated with GC. The abscissa indicates the sample number, and the ordinate represents the differentially expressed gene. The top right histogram is the color gradation. Each rectangle corresponds to a sample expression value. Red indicates high expression and green indicates low expression. **b** Binding sites between LINC01314 and KLK4-3′UTR predicted using the biological prediction website. **c** Luciferase activity detected using dual-luciferase reporter gene assay. *p < 0.05, compared with the NC group. *GC* gastric cancer, *KLK4* kallikrein 4, *lncRNA* long non-coding RNA, *UTR* untranslated region, *Wt* wild type, *Mut* mutant

Firstly, microarray data analysis was conducted to screen the GC-related differentially expressed genes (DEGs). Analysis of GC chip data GSE19826 revealed that LINC01314 was poorly expressed in GC (Fig. 1a). Therefore, LINC01314 was selected for the current study. Additionally, the MEM website suggested that KLK4 gene was the target of LINC01314 and may regulate the Wnt signaling pathway (Table 2). Furthermore, the biological prediction website RNA22 (https://cm.jefferson.edu/rna22/) predicted the existence of a specific binding site between the KLK4 gene sequence and LINC01314 sequence (Fig. 1b). Subsequently, KLK4 was identified to be a target gene of LINC01314 (Fig. 1c) by dual-luciferase reporter gene assay. The results revealed that compared with the NC group, the luciferase activity of wild type (Wt)-KLK4 was attenuated in the OE-LINC01314 group (*p* < 0.05) while that of Mut-KLK4 did not differ greatly (*p* > 0.05). These results demonstrate that LINC01314 could specifically bind to KLK4 gene.

**Table 2:** KEGG analysis for the target gene of LINC01314

Pathway	Gene
Neuroactive ligand-receptor interaction	*CHRNB2; LTB4R; F2; GRIK5; GRM5; OPRD1*
Primary bile acid biosynthesis	*SLC27A5; BAAT*
Glutamatergic synapse	*SLC38A3; GRIK5; GRM5*
Taurine and hypotaurine metabolism	*BAAT*
Bile secretion	*SLC27A5; BAAT*
Glycosphingolipid biosynthesis	*ST8SIA5*
Biosynthesis of unsaturated fatty acids	*BAAT*
Glycosaminoglycan biosynthesis—heparan sulfate/heparin	*EXTL1*
Proximal tubule bicarbonate reclamation	*SLC38A3*
Wnt signaling pathway	*KLK4*

### LINC0134 inhibits the Wnt signaling pathway by down-regulating KLK4

**Fig. 2:**
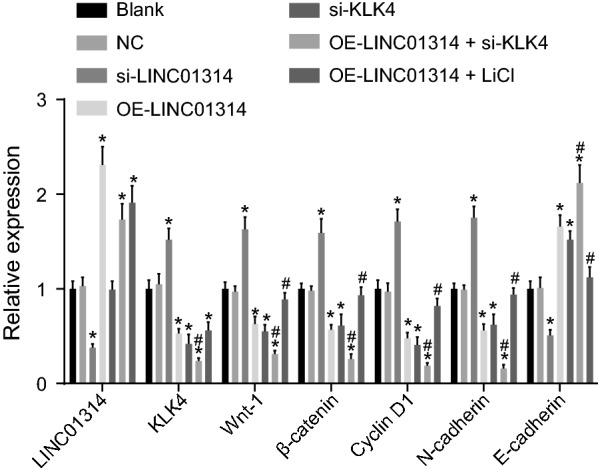
The mRNA levels of KLK4, Wnt-1, β-catenin, cyclin D1 and N-cadherin were decreased while those of E-cadherin increased with elevation of LINC01314; *p < 0.05 vs. the blank group; ^#^*p *< 0.05 vs. the OE-LINC01314 group; RT-qPCR, reverse transcription quantitative polymerase chain reaction. *KLK4* kallikrein 4

**Fig. 3:**
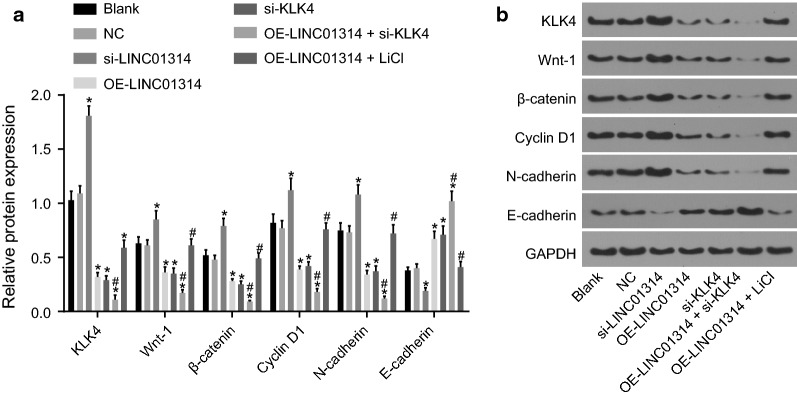
Over-expressed LINC0134 could inhibit the activation the Wnt/β-catenin signaling pathway by inhibiting the expression of KLK4. **a** The protein levels of KLK4, Wnt-1, β-catenin, cyclin D1, N-cadherin and E-cadherin detected by Western blot analysis; **b** the grey value of KLK4, Wnt-1, β-catenin, cyclin D1, N-cadherin, E-cadherin, and GAPDH determined by Western blot analysis; *p < 0.05 vs. the blank group; ^#^*p *< 0.05 vs. the OE-LINC01314 group. *NC* negative control, *KLK4* kallikrein 4, *GAPDH* glyceraldehyde-3-phosphate dehydrogenase

We performed RT-qPCR (Fig. 2) and Western blot analysis (Fig. 3a, b) to evaluate the expression of LINC01314, Wnt-1, β-catenin, cyclin D1, N-cadherin and E-cadherin in all groups, in order to elucidate the mechanism of LINC01314 in GC. The results of RT-qPCR showed that compared with the blank group, the si-LINC01314 group presented with decreased expression of LINC01314 (*p *< 0.05), while the OE-LINC01314 group, the OE-LINC01314 + si-KLK4 group, and the OE-LINC01314 + LiCl group exhibited increased expression of LINC01314 (all *p *< 0.05). Also, the blank group, NC group and si-KLK4 group showed no significant differences in the expressions of LINC01314 with *p* values > 0.05. Furthermore, when compared with the blank group, the si-LINC01314 group showed notably elevated mRNA levels of KLK4, Wnt-1, β-catenin, cyclin D1 and N-cadherin (all *p *< 0.05), while significantly reduced mRNA levels of E-cadherin (*p* < 0.05), which was opposite to the results observed in the OE-LINC01314, si-KLK4, and OE-LINC01314 + si-KLK4 groups (all *p* < 0.05). Compared with those in the OE-LINC01314 group, the mRNA levels of KLK4, Wnt-1, β-catenin, cyclin D1 and N-cadherin in the OE-LINC01314 + si-KLK4 group were found to be markedly down-regulated (all *p *< 0.05) while the mRNA level of E-cadherin was elevated significantly (all *p *< 0.05). Relative to the OE-LINC01314 group, the OE-LINC01314 + LiCl displayed elevated expression of KLK4, Wnt-1, β-catenin, cyclin D1 and N-cadherin while that of E-cadherin was reduced (all *p* < 0.05) No evident differences were found in the mRNA levels of KLK4, Wnt-1, β-catenin, cyclin D1, N-cadherin and E-cadherin among the blank group and the NC group (all *p *> 0.05).

The results of Western blot analysis demonstrated that the si-LINC01314 group exhibited increased protein levels of KLK4, Wnt-1, β-catenin, cyclin D1 and N-cadherin compared to the blank group and the si-LINC01314 group (all *p *< 0.05), while those of E-cadherin were significantly decreased (all *p *< 0.05), opposite to the tendency in the OE-LINC01314, si-KLK4, and OE-LINC01314 + si-KLK4 groups (all *p* < 0.05). Compared with those in the OE-LINC01314 group, the protein levels of KLK4, Wnt-1, β-catenin, cyclin D1 and N-cadherin in the OE-LINC01314 + si-KLK4 group were significantly reduced (all *p *< 0.05), while the protein levels of E-cadherin were increased evidently (all *p *< 0.05), whereas opposite trends were observed in the OE-LINC01314 + LiCl group (all *p* < 0.05). No remarkable differences were found in the protein levels of KLK4, Wnt-1, β-catenin, cyclin D1, N-cadherin and E-cadherin among the blank group and the NC group (all *p *> 0.05). Taken together, the aforementioned results suggest that up-regulating LINC0134 could inhibit the Wnt signaling pathway by reducing the protein levels of KLK4, Wnt-1, β-catenin, cyclin D1 and N-cadherin.

### Up-regulation of LINC01314 or down-regulation of KLK4 suppresses GC cell invasion

**Fig. 4:**
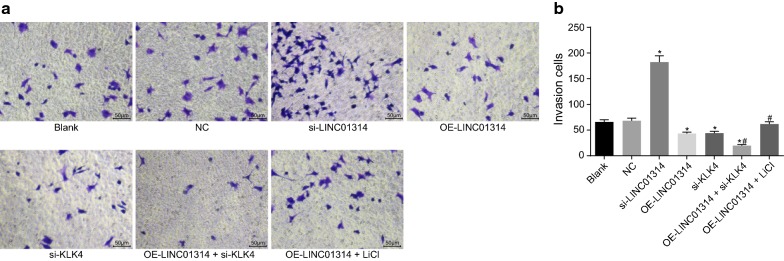
Over-expressed LINC01314 inhibited cell invasion in GC. **a** The cells penetrating through the matrigel stained with crystal violet (×200). **b** statistical results of the number of the cells penetrating through the matrigel; *p < 0.05 vs. the blank group; ^#^*p *< 0.05 vs. the OE-LINC01314 group. *NC* negative control, *GC* gastric cancer

Transwell assay was conducted in order to assess the effect of LINC01314 on the invasion of GC cells (Fig. 4a, b). After 48 h of cell culture, the number of invasive cells was found to be significantly increased in the si-LINC01314 group (*p* < 0.05) but decreased in the OE-LINC01314, si-KLK4, and OE-LINC01314 + si-KLK4 groups (all *p* < 0.05) in comparison with the blank group. The OE-LINC01314 + si-KLK4 group exhibited reduced number of invasive cells while the OE-LINC01314 + LiCl group exhibited an elevated number of invasive cells relative to the OE-LINC01314 group (all *p* < 0.05). No evident differences in the cell number were found among the blank, NC, and OE-LINC01314 + LiCl groups (*p* > 0.05). The above findings suggested that LINC01314 elevation or KLK4 depletion could significantly reduce invasion of GC cells.

### Up-regulation of LINC01314 or down-regulation of KLK4 inhibits GC cell migration

**Fig. 5:**
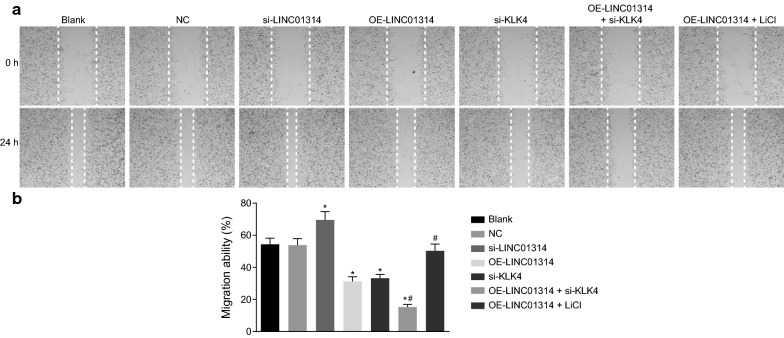
Over-expressed LINC01314 repressed cell migration in GC. **a** Wound-healing condition of GC cells at the 0th and 24th h; **b** histogram of migration ability of GC cells; *p < 0.05 vs. the blank group; ^#^*p *< 0.05 vs. the OE-LINC01314 group. *NC* negative control, *GC* gastric cancer

Furthermore, Scratch test was performed in order to investigate the effect of LINC01314 on migration of GC cells (Fig. 5a, b). A significant increase in GC migration was noted in the si-LINC01314 group, while the opposite trend was observed in the OE-LINC01314, si-KLK4, and OE-LINC01314 + si-KLK4 groups when compared with the blank group after 48 h of cell culture (*p* < 0.05). Meanwhile, consistent with GC cell penetration, GC cell migration was notably attenuated in the OE-LINC01314 + si-KLK4 group in comparison with the OE-LINC01314 group (*p* < 0.05); however, GC cell migration was enhanced in the OE-LINC01314 + LiCl group in contrast to the OE-LINC01314 group (*p* < 0.05). The blank, NC, and OE-LINC01314 + LiCl groups showed no significant differences in terms of cell migration (*p* > 0.05). These findings demonstrated that up-regulation of LINC01314 or down-regulation of KLK4 could inhibit cell migration of GC.

### Up-regulation of LINC01314 or down-regulation of KLK4 regulates factors related to migration and invasion

**Fig. 6:**
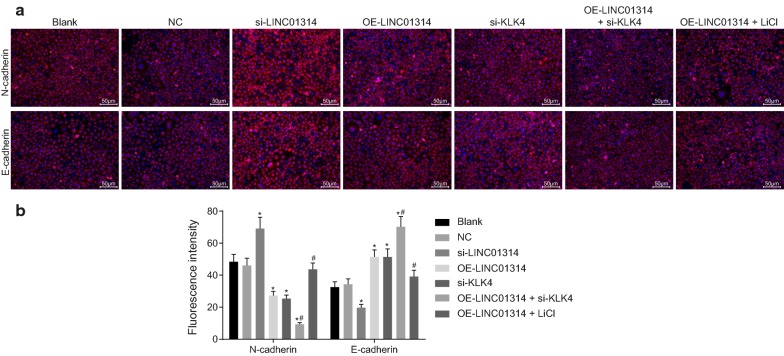
Over-expressed LINC01314 regulated factors related to migration and invasion in GC cells by down-regulating the expression of KLK4. **a** Immunofluorescence staining images of N-cadherin and E-cadherin after treatment; **b** the positive expression rate of N-cadherin and E-cadherin after treatment; *p < 0.05 vs. the blank group; ^#^*p *< 0.05 vs. the siRNA group. *NC* negative control, *KLK4* kallikrein 4

In order to investigate the association between LINC01314 and migration and invasion of GC cells, we evaluated the positive expression rate of N-cadherin and E-cadherin using immunofluorescence (Fig. 6a, b). Compared with the blank group, the positive expression rate of N-cadherin was found to be increased while that of E-cadherin was decreased in the si-LINC01314 group (*p* < 0.05); however, the positive expression rate of N-cadherin was decreased whereas that of E-cadherin was increased in the OE-LINC01314, si-KLK4, and OE-LINC01314 + si-KLK4 groups, relative to the blank group (all *p* < 0.05). In comparison with the OE-LINC01314 group, decreased positive expression rate of N-cadherin and increased that of E-cadherin were observed in the OE-LINC01314 + si-KLK4 group (*p *< 0.05). The OE-LINC01314 + LiCl group exhibited an elevated positive expression rate of N-cadherin and reduced positive expression rate of E-cadherin, while opposite trends were observed in the OE-LINC01314 group (*p* < 0.05). No evident differences were observed in the positive expression rate of N-cadherin and E-cadherin among the blank group, the NC group, and the OE-LINC01314 + LiCl group (*p *> 0.05). Taken together, these findings indicate that LINC01314 elevation could inhibit cell invasion and migration by depleting the expression of KLK4.

### Up-regulation of LINC01314 or down-regulation of KLK4 suppresses tumor growth in nude mice

**Fig. 7:**
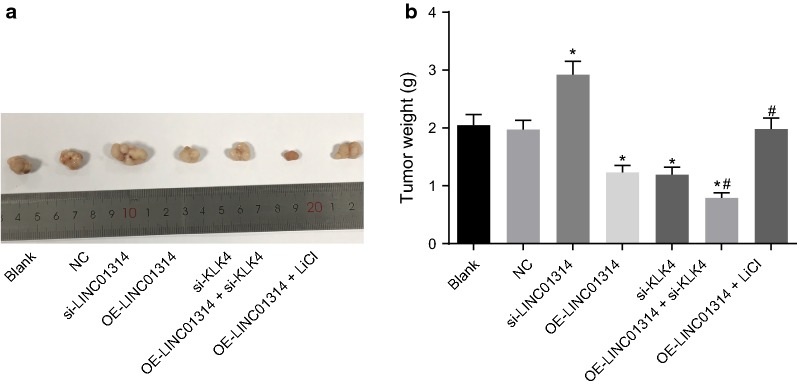
Over-expressed LINC01314 or down-regulated KLK4 suppressed tumor growth in nude mice. **a** Representative tumors excised from the nude mice; **b** the tumor weight of mice; *p < 0.05 vs. the blank group; ^#^*p *< 0.05 vs. the OE-LINC01314 group. *NC* negative control, *GC* gastric cancer, *KLK4* kallikrein 4

Treated GC cells were injected into the nude mice to verify how LINC01314 affected tumorigenicity of GC cells in vivo (Fig. 7a, b). After tumor latency of about 7 days, visible tumors growth was noted at all the inoculation sites of nude mice. In addition, small subcutaneous nodules were observed at the site of inoculation, which initially presented with an elliptical surface, gradually becoming irregular, indicating a 100% tumor formation rate. Compared to the blank group, the weight of tumor in the si-LINC01314 group was noted to be increased (*p* < 0.05), while the OE-LINC01314, si-KLK4, and OE-LINC01314 + si-KLK4 groups displayed reductions in the weight of tumors in nude mice (*p* < 0.05). Compared with the OE-LINC01314 group, tumor weight was found to be reduced in the OE-LINC01314 + si-KLK4 group but elevated in the OE-LINC01314 + LiCl group (all *p* < 0.05). No evident differences were observed in the tumor weight in the blank group, the NC group, and the OE-LINC01314 + LiCl group (*p *> 0.05). These findings showed that LINC01314 elevation or KLK4 depletion could inhibit tumor growth in nude mice with GC cell inoculation.

### Up-regulation of LINC01314 or down-regulation of KLK4 decreases the MVD of tumor in nude mice

**Fig. 8:**
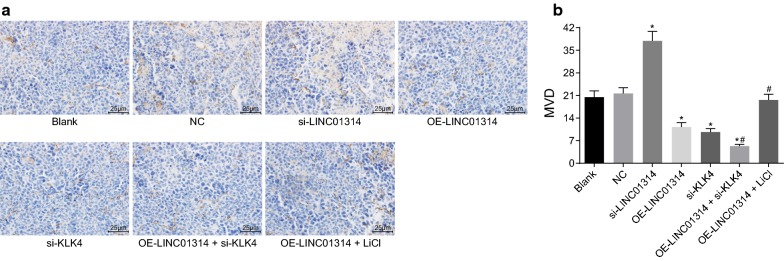
Elevated LINC01314 or depleted KLK4 decreased the MVD of tumor in nude mice (×400). **a** Immunohistochemical staining of CD34; **b** the MVD of mice; *p < 0.05 vs. the blank group; ^#^*p *< 0.05 vs. the OE-LINC01314 group. *NC* negative control, *GC* gastric cancer, *MVD* micro-vessel density, *KLK4* kallikrein 4, *CD34* cluster of differentiation 34

The MVD in transplanted tumors of nude mice was measured, and the results are shown in Fig. 8a, b. The anti-CD34 monoclonal antibody was employed to label the vascular endothelial cells (VECs), and CD34 was found to be located in the cytoplasm of VECs. Significant increases in the MVD of transplanted tumors were observed in the si-LINC0131 group while opposite trends were noted in the OE-LINC01314, si-KLK4, and OE-LINC01314 + si-KLK4 groups in contrast to the blank group (*p* < 0.05). Compared with the OE-LINC01314 group, the MVD of transplanted tumors was reduced in the OE-LINC01314 + si-KLK4 group, while elevations were noted in the OE-LINC01314 + LiCl group (all *p* < 0.05). No significant differences were noted among the blank, NC, and OE-LINC01314 + LiCl groups (*p* > 0.05). All in all, LINC01314 over-expression or KLK4 silencing could reduce the MVD of tumor in nude mice with GC.

### Up-regulation of LINC01314 or down-regulation of KLK4 suppresses angiogenesis of GC

**Fig. 9:**
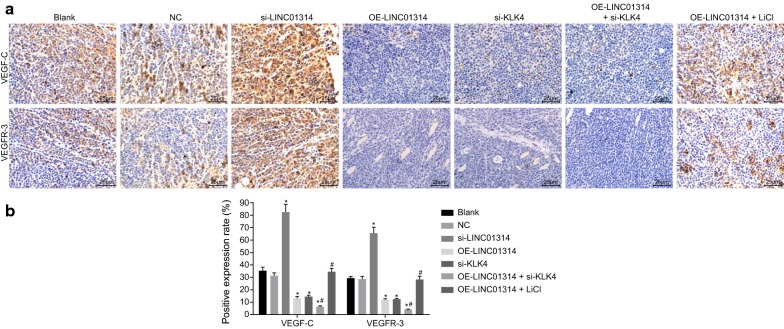
Over-expressed LINC01314 or KLK4 silencing repressed angiogenesis of GC. **a** Immunohistochemical staining of VEGF-C and VEGFR-3; **b** the positive expression rate of VEGF-C and VEGFR-3; *p < 0.05 vs. the blank group; ^#^*p *< 0.05 vs. the OE-LINC01314 group. *NC* negative control, *GC* gastric cancer, *KLK4* kallikrein 4, *VEGF* vascular endothelial growth factor, *VEGFR* vascular endothelial growth factor receptor

Immunohistochemistry was performed to detect the positive expression rate of VEGF-C and VEGFR-3 in order to investigate the role of LINC01314 in angiogenesis of GC (Fig. 9). Relative to the blank group, there were significant elevations in both VEGF-C and VEGFR-3 protein expression in the si-LINC01314 group, while decreased VEGF-C and VEGFR-3 protein expression was noted in the OE-LINC01314, si-KLK4, and OE-LINC01314 + si-KLK4 groups (all *p* < 0.05). Compared with the OE-LINC01314 group, the OE-LINC01314 + si-KLK4 group exhibited down-regulated protein expression of VEGF-C and VEGFR-3 while that in the OE-LINC01314 + LiCl group was found to be up-regulated (all *p* < 0.05). Lastly, no significant differences were noted in the blank, NC, and OE-LINC01314 + LiCl groups (all *p* > 0.05). The above findings demonstrated that LINC01314 elevation or KLK4 depletion could suppress angiogenesis in GC.

## Discussion

GC accounts for the majority of the cancer-related mortality in Asian countries; however, the cascade of events occurring during its pathogenesis remains to be fully understood [26]. LncRNAs have been established to be crucial in the regulation of numerous cellular processes including cell proliferation, differentiation, migration, invasion, and autophagy, and particularly, GC tumorigenesis and progression [27]. The current study aimed to investigate the effects of LINC01314 on regulating cell invasion and migration of GC cells, and uncovered that LINC01314 elevation could inhibit the migration, invasion, and angiogenesis of GC cells.

Firstly, LINC01314 was noted to be poorly expressed in human GC cells. Accumulating studies have demonstrated the involvement of lncRNAs in various biological pathways and tumor development, metastasis and in the inactivation of main tumor-suppressors [28]. Additionally, deregulation of lncRNAs was noted to contribute to the progression of various human tumors by inducing abnormal gene expressions [29]. For example, Sun et al. revealed that lncRNA GAS5 expression was down-regulated in GC tissues, and was further correlated with poor prognosis and overall survival rate of GC patients [30]. Furthermore, lncRNA HMlincRNA717 expression was poorly expressed in both GC cell lines and tissues, and was suggested to serve as a new diagnostic and prognostic marker for GC patients owing to its implications in cancer occurrence and progression [31].

Additionally, the current study demonstrated that over-expression of LINC01314 inhibited cell invasion and migration of GC cells via inactivation of the Wnt/β-catenin signaling pathway by negatively targeting KLK4. The present study provided evidence that KLK4 was a target gene of LINC01314. Recently, multiple microRNAs (miRs) have been identified to regulate the expression of KLK at a post-transcriptional level, and exploration of the target interaction between miRs and KLK4 could improve the prognosis of human diseases [32]. For instance, miR-378, could bind to KLK4 and thus participate in the development of prostate cancer as reported by a previous study [33]. In addition, lncRNA ASLNC04080 was demonstrated to be correlated to KLK3, which could serve as a diagnostic marker for cancers [34]. Furthermore, it has been previously verified that depletion of KLK4 could inhibit the cell proliferation and growth in OSCC via inactivation of the Wnt/β-catenin signaling pathway, accompanied by down-regulated Wnt-1, β-catenin, and cyclinD1 [15], which was consistent with the results of the current study. Tang et al. [35] emphasized the key role of Wnt-1 in the antitumor effects of diallyl disulfide on GC cell apoptosis. Additionally, a previous study focusing on several human cancers including GC reported that the expression of cyclin D1 gene was also notably high in GC, and targeting this gene using specific inhibitory compounds may serve as another effective treatment for GC [36]. More importantly, lncRNAs with defective expressions have been implicated to abnormally trigger the Wnt/β-catenin signaling pathway leading to subsequent deregulated activity of downstream targets [19]. The work of Li and colleagues, for example, demonstrated that over-expressing VGLL4 can suppress migration and invasion of GC cells, and that it can also suppress epithelial-mesenchymal transition by down-regulating the Wnt/β-catenin signal pathway [37]. These previous works supported the possible crosstalk between the Wnt/β-catenin signaling events and the role of LINC01314, with the latter almost certainly acting as a suppressor of the former during GC development.

The succeeding experiments showed that elevated LINC01314 or depleted KLK4 could inhibit angiogenesis in GC cells by negatively regulating the Wnt/β-catenin signaling pathway. KLKs, as a subunit of secreted serine proteases found in various tissues and cells, are critical for diverse pathophysiological progressions, including angiogenesis [38]. Furthermore, KLK4 depletion was revealed to lead to suppressed cell migration and invasion in OSCC due to inactivation of the PI3K/AKT signaling pathway [39]. Previous microarray experiments revealed that some aberrant lncRNA expressions in GC were related to clinical pathological process such as increased tumor size, poor differentiation, and LNM [40]. Additionally, E-cadherin has been shown to exhibit tumor suppressing activity, and was further associated with LNM [41], while N-cadherin over-expression in tumor cells permits lympho-vascular invasion of tumor cells which is associated with the gain of metastatic ability of cancer cells like GC [42]. Angiogenesis, as a common characteristic of all cancers, is related to tumor grade and malignancy, and is also crucial in tumor progression [43]. Notably, a previous study reported that the Wnt/β-catenin signaling pathway can regulate angiogenesis, infiltration and metastasis by regulating the expression of angiogenic factors in hepatocellular carcinoma cell [44]. Taken together, we can conclude that LINC01314 over-expression could decrease N-cadherin, VEGF-C and VEGFR-3 levels, while increasing E-cadherin expressions, thus leading us to speculate that it can also inhibit migration, invasion and angiogenesis in GC.

## Conclusion

In conclusion, the findings of the current study demonstrated that LINC01314 can inhibit GC cell proliferation, migration, invasion and angiogenesis through suppression of the Wnt/β-catenin signaling pathway by down-regulating KLK4. Thus, LINC01314 can serve as a novel therapeutic target for GC tumor suppression and may serve as a reference for innovative GC treatment modalities. It is also recommended that a in vivo experiment with a larger sample size should be conducted in further studies to completely understand the underlying mechanism of LINC01314 in GC.

## Abbreviations

GC: gastric cancer
LNM: lymph node metastasis
DEGs: differentially expressed genes
lncRNAs: long non-coding RNAs
RIPA: Radio Immunoprecipitation Assay
PVDF: polyvinylidene fluoride
SDA-PAGE: sodium dodecyl sulfate polyacrylamide gel electrophoresis
